# Rootstock genotype shapes whole-plant 3-D architecture and biomass allocation in field-grown grapevines

**DOI:** 10.1093/aob/mcaf193

**Published:** 2025-09-01

**Authors:** Lukas Fichtl, Katharina Steng, Andrea Schnepf, Matthias Friedel

**Affiliations:** Department of General and Organic Viticulture, Hochschule Geisenheim University, 65366 Geisenheim, Germany; Department of General and Organic Viticulture, Hochschule Geisenheim University, 65366 Geisenheim, Germany; Institute of Bio- and Geoscience: Agrosphere (IBG-3), Forschungszentrum Juelich GmbH, 52428 Juelich, Germany; Department of General and Organic Viticulture, Hochschule Geisenheim University, 65366 Geisenheim, Germany

**Keywords:** Perennials, *Vitis vinifera*, grafting, root system architecture, canopy architecture, digital phenotyping, biomass allocation, viticulture, vineyard establishment

## Abstract

**Background and Aims:**

In perennial crops, efficient resource acquisition critically depends on whole-plant architecture, encompassing both canopy and root systems. In grafted grapevine, research has largely focused on scion canopy structure, whereas root system architecture – despite its key role in water and nutrient uptake – remains underexplored. This study comprehensively analysed whole-plant 3-D architecture during vineyard establishment, investigating how different rootstock genotypes influence both root and shoot development.

**Methods:**

Riesling scions were grafted onto three rootstock genotypes (101-14, SO4 and 110R) and planted in a vineyard following a randomized complete block design. Whole-plant excavations and high-resolution 3-D digitization were performed to capture spatial data of root and shoot systems from 96 vines at four time points over 2 years (3, 6, 15 and 18 months after planting). Key architectural parameters and biomass partitioning were quantified.

**Key Results:**

Rootstock genotype strongly influenced whole-plant 3-D architecture and biomass allocation. 110R developed significantly deeper, vertically oriented root systems (max depth 180 cm) and exhibited higher root-to-shoot biomass ratios compared to SO4 and 101-14. Multivariate analysis identified deep root length and overall spatial root system dimensions as primary discriminators among genotypes. Root growth across all genotypes was spatially biased along the planting row, with limited extension into the inter-row soil.

**Conclusions:**

Rootstock genotype is a key determinant of whole-plant 3-D architecture and biomass partitioning. The integration of above- and below-ground structural data enables mechanistic interpretation of rootstock-mediated traits relevant to resource acquisition and stress adaptation. Our comprehensive 3-D data set provides a valuable foundation for functional–structural plant modelling and offers practical insights for targeted breeding and management strategies to enhance climate resilience in perennial crops.

## INTRODUCTION

The efficiency of resource acquisition in agricultural cropping systems is closely linked to the spatiotemporal dynamics of plant architecture, which governs crop performance, adaptability, yield potential and product quality (Reinhardt and Kuhlemeier, 2002). In perennial crops such as grapevine (*Vitis vinifera* L.), predominantly cultivated as grafted plants, the integration of rootstock and scion traits significantly shapes whole-plant architecture and function, with rootstocks strongly influencing scion growth and adaptability to abiotic constraints (Zhang et al., 2016; Ollat et al., 2018; Fichtl et al., 2023). By combining distinct rootstock and scion genotypes, grafting offers a strategic approach to tailor root and shoot traits to specific environmental conditions, enhancing the plant’s overall performance and the sustainability of cropping systems (Delrot et al., 2020). Such trait optimization through grafting has become increasingly critical in the context of climate change, as intensified abiotic stresses require improved resource capture under fluctuating environmental conditions (Ollat et al., 2018; Lynch, 2022). Therefore, a holistic understanding of whole-plant architecture is essential, recognizing the interplay between above- and below-ground structures that collectively determine plant adaptability and resilience. Above-ground, canopy architecture governs light interception, microclimate dynamics, disease susceptibility and fruit quality (Smart et al., 1990; Haselgrove et al., 2000; Louarn et al., 2007). Below-ground, root system architecture (RSA) determines the spatial and temporal acquisition of water and nutrients, especially in heterogeneous soil environments (Fitter, 1987; Osmont et al., 2007; Wasson et al., 2012; White et al., 2013; Lynch, 2022). Although the significance of RSA for plant performance is generally acknowledged, its detailed characterization in grafted perennial crops under field conditions remains underexplored (Roumet et al., 2006; Freschet et al., 2021). Recent studies on grapevine architecture have delivered detailed investigations of scion canopy architecture traits, yielding robust quantitative models for predicting light interception and growth patterns (Louarn et al., 2007; Schmidt et al., 2022). To date, however, no studies have investigated grapevine RSA in comparable detail under field conditions. RSA modulates scion development and is particularly critical under drought conditions, where a functional balance between shoot vigour and RSA is essential to match transpirational demand with water uptake capacity (Carbonneau, 1985; Soar et al., 2006; Alsina et al., 2011; Marguerit et al., 2012; Tardieu, 2012; Zhang et al., 2016). Integrating both above- and below-ground architectural data is therefore key to developing predictive models of resource acquisition and biomass allocation.

Such predictive models are particularly critical for understanding resource uptake, especially water, during the vulnerable phase of vineyard establishment (Gutiérrez-Gamboa et al., 2021; Fichtl et al., 2023). This early establishment period is not only a high-risk window for plant survival under intensifying climate pressure (Gutiérrez-Gamboa et al., 2021), but it also represents a formative stage during which root and shoot systems co-develop and lay the structural foundation for long-term vine performance (Larrey et al., 2025).

This study investigates how rootstock genotype controls the integrated development of grapevine root and shoot systems during vineyard establishment. We selected three widely used and well characterized rootstock genotypes – ‘101-14 Millardet et de Grasset’, ‘Selection Oppenheim 4’, and ‘Richter 110’ – that differ markedly in rooting depth, drought tolerance profiles and genetic background (Ollat et al., 2014; Zhang et al., 2016; Fort et al., 2017). Previous greenhouse studies have documented architectural differences among these rootstocks under controlled conditions, particularly in rooting depth distribution, root production and elongation dynamics (Fort et al., 2017; Cuneo et al., 2021). However, it remains unclear whether such traits are equally expressed under field conditions. Therefore, high-resolution field phenotyping is essential to complement findings from controlled environments, providing data that can be used to develop robust growth models. These models, in turn, are crucial for predicting rootstock performance across diverse environmental and management scenarios.

Specifically, we aim to (i) deepen the mechanistic understanding of genotype-driven whole-plant architecture and biomass allocation in field-grown grapevines; (ii) derive practical implications for rootstock choice and early vineyard management; and (iii) provide high-resolution, spatially explicit data to support genotype-specific parameterization of root growth and allocation models in woody perennials. By addressing these aims under realistic field conditions and integrating 3-D–resolved root and shoot data, our study contributes both mechanistic insight and application-oriented value to perennial crop research in the face of climate change.

## MATERIALS AND METHODS

### Plant material and experimental design

The field experiments were conducted at the vineyards of the Department of General and Organic Viticulture, Hochschule Geisenheim University, Germany (lat. 49°59′16″N, long. 7°56′56″E). A new vineyard was established in May 2023 with grafted vines of the scion variety ‘Riesling’ (*Vitis vinifera* L.; clone N90), grafted onto three distinct rootstock varieties: ‘101-14 Millardet et de Grasset’ [‘101-14’] (*Vitis riparia* × *Vitis rupestris*; clone 3), ‘Selection Oppenheim 4’ [‘SO4’] (*Vitis berlandieri* × *Vitis riparia*; clone 31 OP), and ‘Richter 110’ [‘R110’] (*Vitis berlandieri* × *Vitis rupestris*; clone 152). These rootstocks were selected based on their contrasting drought response and rooting profiles: 110R is widely characterized as deep-rooting and drought-tolerant (Carbonneau, 1985; Smart et al., 2006; Dry, 2007; Keller, 2020; Bartlett et al., 2022), 101-14 as shallow-rooting and drought-sensitive (Smart et al., 2006; Dry, 2007; Fort et al., 2017; Bartlett et al., 2022), whereas SO4 serves as an intermediate reference and represents one of the most commonly planted rootstocks in Germany and other temperate viticultural regions (Schmid et al., 2009; Ollat et al., 2015). All vines were sourced from the same grapevine nursery (DLR Rheinpflalz) and propagated under uniform conditions. The 1-year-old vines, with adventitious roots pruned to approximately 10–15 cm, were planted according to practical standards, using a GPS-supported mechanical planting machine and maintaining an inter-vine distance of 1 m and a row width of 2 m.

The main experiment was designed as a fully randomized, complete block design with four blocks, eight replicates per rootstock genotype, and ten vines per replicate. A metal-free trellis system, comprising wood posts, bamboo planting sticks and plastic wires, was employed to avoid electromagnetic interference during 3-D digitization. Additionally, a side experiment was initiated in May 2024 in close proximity to the main experiment. This included an extra row where cuttings of the same Riesling clone, grafted onto SO4 and R110, were planted with five replicates per genotype and five vines per replicate, specifically to study new shoot and root production during the initial weeks of vineyard establishment.

Vineyard management practices adhered strictly to organic viticulture principles throughout the study. The plot was left fallow for 6 years before planting, and a diverse cover crop was incorporated into the soil. No fertilizers were applied before or during the experiment. The trial was conducted under dry-farmed conditions without irrigation. In both growing seasons, shoot thinning was conducted to maintain only one shoot per vine. Following the initial growing season, shoots were pruned back to two nodes per vine and subsequently thinned again to a single shoot per vine in the second season, while lateral shoots were preserved. Soil and weed management was performed mechanically and a natural grass/clover cover crop was grown in every other row.

### Soil sampling and analysis

The vineyard site is characterized by deep, well-structured soils formed from loess substrates. The soil profile consists of sandy loam over loamy sand to strongly sandy loam (loess), underlain by deep, carbonate-bearing, moderately gravelly sands originating from Pleistocene deposits or marine sands of the Alzey Formation (HLNUG, 2025). At the onset of the experiment (May 2023), disturbed soil samples were collected for chemical analyses. A total of 16 mixed samples were obtained from eight locations (two per block, with one sample taken from under the vine and one from the in-row position) at two depth intervals (0–30 and 30–60 cm). Disturbed samples were collected using a Pürckhauer soil sampler, with each mixed sample comprising four drillings per block × position combination. Soil organic matter (SOM), total carbon and nitrogen were quantified using the Dumas method, whereas soil organic carbon (SOC) was determined by subtracting carbonate carbon (measured according to Scheibler) from total carbon. In addition, undisturbed soil samples were collected in July 2024 to assess bulk density as a proxy for soil compaction. For these, one trench per block was excavated and undisturbed samples were taken at three depths (30, 60 and 90 cm) from two locations (under the vine and beneath the tractor traffic lane). Stainless steel metal rings were hammered into the freshly exposed soil profile with a sampling head, the cores were extracted, and excess soil was removed with a sharp knife. Bulk density was calculated as the ratio of the dry weight of the sample to its volume (100 cm^3^).

### Soil moisture and weather monitoring

The maximum plant-available soil water is estimated at 400–440 mm (HLNUG, 2025). Soil moisture monitoring was performed by measuring the volumetric water content using a capacitance sensor (Diviner 2000, Sentek Pty Ltd, Stepney, SA, Australia). Two months after the beginning of the experiment (July 2023), eight Diviner access tubes were installed (two per block) in the inter-vine space to a depth of 1.60 m, with data recorded at 10 cm intervals. Measurements were conducted weekly during the growing season (April–October) and fortnightly during winter and vine dormancy. The mean relative deviation of soil moisture at each depth was calculated by normalizing the observed soil moisture against the maximum recorded value at that depth across the study period. Continuous weather data, including air temperature at 2 m height and daily precipitation, were obtained from a nearby weather station (approximately 400 m distance) provided by the Hochschule Geisenheim University weather station network (HGU, 2025).

### Plant architecture measurements

Within the main experiment, the whole-plant architecture of 96 grapevines was measured at four time points within the first two years of vineyard establishment. At each designated time point, eight grapevines of each rootstock genotype (one per replicate) were assessed, resulting in measurements of 24 vines at 3 months (T1: July 2023), 6 months (T2: November 2023; after leaf fall), 15 months (T3: July 2024) and 18 months (T4: November 2024; after leaf fall) after planting. To ensure natural competitive dynamics among root systems, only every second grapevine in each replicate was selected for measurements, with the additional criteria that each selected vine was vital as well as flanked by two healthy neighbouring vines.

In the side experiment, both below- and above-ground growth was monitored weekly over a 5-week period following planting. Each week, measurements of early root and shoot growth were taken from 10 vines (five per genotype) to capture early shoot and root growth dynamics.

#### Shoot and leaf digitization

In the main experiment, plant architecture was captured through 3-D digitization, employing a Fastrak 3-D digitizer (Polhemus, Colchester, USA). This equipment, based on electromagnetic principles, consists of a main unit, a transmitter and a pointer. The transmitter generates a low-frequency electromagnetic field, facilitating the recording of spatial coordinates at the pointer tip. Functional annotation of these coordinates was conducted in real-time using DigiTool software (customizable research software; Moualeu-Ngangué et al., 2020), which permits the direct assignment of topological information to each captured point.

Vine shoots and leaves were digitized post-cutting at the grafting point. Shoots were suspended in a hanging state during digitization, with the transmitter positioned approximately 30 cm from the specimen. The above-ground plant architecture was captured following the protocol described by Schmidt et al. (2019): digitization began at the grafting point (point 0) and proceeded to the shoot initiation point (point 1), with each subsequent node systematically recorded by marking the leaf axis; lateral shoots were digitized similarly and linked directly to their respective mother nodes. A six-point scheme was used for leaf digitization to enable leaf area estimation: PL1 was assigned at the petiole base in the direction of the shoot apex, PL2 at the adaxial leaf base, PL3 at the junction of the midrib with the veins spanning the central lobe (adaxial side), PL4 at the midrib tip, and PL5 and PL6 at the tips of the left and right veins spanning the central lobe, respectively (Schmidt et al., 2019). Apical nodes of primary or lateral shoots shorter than 1 cm and leaves with a primary vein length under 3 cm were excluded from digitization.

#### Root system excavation and digitization

RSA was digitized following the pipeline outlined by Fichtl et al. (2024). Briefly, the root systems of individual grapevines were carefully excavated to minimize loss and damage, using manual techniques and small handheld tools. The grapevines, initially established as rooted cuttings, were planted such that the base of the stem was about 20 cm below the soil surface. Excavation commenced with the removal of the top 10 cm of soil over a 1 m^2^ area and the digging of a trench around 1 m from the vine to a depth of approximately 1.50 m. A horizontal wooden framework was used to stabilize the vine at its grafting point during excavation. Root systems were then meticulously exposed including all lateral and fine roots, primarily using hand tools and occasionally a weeding trowel for loosening compact soil, and then gently brushed to reveal individual roots. The entire root structure was secured and aligned to reflect its natural orientation within the soil.

Following excavation, 3-D digitization was performed using the same technology as for above-ground digitization (Polhemus Fastrak System). A custom frame was positioned approximately 60 cm above the soil to suspend the transmitter, ensuring the electromagnetic field targeted the exposed root system accurately. Digitization commenced at the grafting point and progressed through systematic points on the stem down to the adventitious roots and their branching, ensuring a comprehensive mapping of root topology (Fichtl et al., 2024).

### 3-D data processing and parameter estimation

The acquired 3-D RSA data were transformed into the interoperable RSML format for parameter estimation and subsequently converted into .vtp format for three-dimensional visualization (Fichtl et al., 2024). Internode lengths were calculated as the Euclidean distances between successive nodes, and single leaf area was estimated based on the digitized lengths of the secondary right and left leaf veins as validated for Riesling leaves (Döring et al., 2013). Global root system characteristic measures – including total root system length, maximum rooting depth, maximum horizontal spread, 3-D convex hull volume and 2-D convex hull areas (e.g. on the XY plane) – were derived using the ArchiDART package in R (version 3.4; Delory et al., 2018). Cumulative root length per predefined soil layer was computed by partitioning the Euclidean length of each line segment evenly across the layers it traversed, as implemented in a custom R function.

RSA was further characterized by three parameters. First, the aspect ratio was calculated as


$$\mathrm{Aspect}\;\mathrm{Ratio}\;=\;\frac{{\mathrm{proj}}_{x}^{\max}-{\mathrm{proj}}_{x}^{\min}}{{\mathrm{proj}}_{y}^{\max}-{\mathrm{proj}}_{y}^{\min}}$$

where ${\mathrm{proj}}_{x}^{\max}$ and ${\mathrm{proj}}_{x}^{\min}$ denote the maximum and minimum *x*-coordinates (inter-row direction) of the 2-D convex hull on the XY plane, and ${\mathrm{proj}}_{y}^{\max}$ and ${\mathrm{proj}}_{y}^{\min}$ are the corresponding *y*-coordinates (planting row direction). Second, a Directional Bias Index (DBI) was computed to quantify the degree to which root length is preferentially allocated in a dominant direction relative to a defined coordinate system centred on the vine, where (0,0) represents the stem. In this framework, the horizontal projection of the 3-D RSA data is partitioned into 10 × 10 cm grid cells, each of which is assigned specific *x*- and *y*-coordinates relative to the vine’s centre; here, the *y*-coordinate represents the vertical (planting row) direction and the *x*-coordinate denotes the horizontal (inter-row) direction. For each vine, grid-aggregated root lengths are used to compute two sums: $L_{\mathrm{vertical}}$, the total root length in grid cells where the *y*-coordinate of the grid cell exceeds that of the *x*-coordinate, and $L_{\mathrm{horizontal}}$, the total root length in grid cells where the absolute value of the grid cell *x*-coordinate is greater than or equal to that of its *y*-coordinate. The DBI is then computed as


$$\mathrm{DBI}\;=\;\frac{L_{\mathrm{horizontal}}-L_{\mathrm{vertical}}}{L_{\mathrm{horizontal}}+L_{\mathrm{vertical}}}\;$$

yielding values in the range from –1 to 1. Under this formulation, a negative DBI indicates that the majority of the root length is oriented in the vertical (planting row) direction, whereas a positive DBI signifies a greater lateral (inter-row) spread. This index complements the geometric measure of the aspect ratio by additionally accounting for the actual distribution of root length across the coordinate-defined grid. Finally, the proportion of root length outside the designated planting area (2 m^2^) was determined as


$$\frac{L_{\mathrm{outside}}}{L_{\mathrm{total}}}$$

where $L_{\mathrm{outside}}$ is the cumulative root length beyond the predefined area boundary (*x* −100 to 100 and *y* −50 to 50 on the XY plane) and $L_{\mathrm{total}}$ is the total root length of the vine.

#### Early root and shoot growth

In the side experiment focused on root initiation and bud break, newly grown shoots were counted and measured with callipers for length. Concurrently, root systems were excavated as outlined before. Root analyses involved counting the number of newly formed root tips and measuring the length of each new root using a calliper.

### Estimation of biomass allocation

For the quantification of biomass allocation within the study, each vine was divided into four compartments: roots, stem, shoots, and leaves. Following whole-plant digitization, the wet weight of each compartment was recorded. Subsequently, samples were oven-dried at a constant temperature of 60 °C for approximately 2 weeks to ensure complete dehydration, after which the dry weights were measured. Mass fractions for the woody compartments (shoot, stem, root) were calculated as:


$$\mathrm{Mass}\;\mathrm{Fraction}=\frac{\mathrm{Dry}\;\mathrm{Weight}\;\mathrm{of}\;\mathrm{Compartment}}{\mathrm{Total}\;\mathrm{Woody}\;\mathrm{Dry}\;\mathrm{Weight}\;\mathrm{of}\;\mathrm{Vine}}$$

Furthermore, specific leaf area (SLA) was determined by dividing the total leaf area (obtained from 3-D digitization) by the leaf dry weight:


$$\mathrm{SLA}=\frac{\mathrm{Total}\;\mathrm{Leaf}\;\mathrm{Area}}{\mathrm{Leaf}\;\mathrm{Dry}\;\mathrm{Weight}}$$

Similarly, specific shoot length (SSL) and specific root length (SRL) were computed as the total shoot length and total root length (derived from 3-D digitization) divided by their corresponding dry weights:


$$\mathrm{SSL}=\frac{\mathrm{Total}\;\mathrm{Shoot}\;\mathrm{Length}}{\mathrm{Shoot}\;\mathrm{Dry}\;\mathrm{Weight}}\;\mathrm{SRL}=\frac{\mathrm{Total}\;\mathrm{Root}\;\mathrm{Length}}{\mathrm{Root}\;\;\mathrm{Dry}\;\mathrm{Weight}}$$

### Data and statistical analysis

Data analysis, including the import of digitized 3-D data, topological reconstruction, segmentation and transformation (e.g. into RSML format), was conducted using R (version 4.4.0; R Core Team, 2024) in combination with the RStudio GUI (version 2024.04; RStudio Team, 2020). Data visualization was performed using the ggplot2 package (version 3.5.1; Wickham, 2016); 3-D data were visualized using ParaView (version 5.12.0; Ahrens et al., 2005). Statistical analyses were carried out in R utilizing the lme4 (version 1.1-35.1) and lmerTest (version 3.1-3) packages (Bates et al., 2015; Kuznetsova et al., 2017) for linear mixed-effects models employed to assess the effects of rootstock genotype, time and their interaction on measured parameters, and block included as a random effect. Normality of residuals was tested via the Shapiro–Wilk test (stats package, version 4.3.0, R Core Team, 2024). In cases where the normality assumption was violated, non-parametric generalized linear mixed models with an appropriate distribution (Gamma) were employed using the lme4 package. Post-hoc pairwise comparisons among rootstock genotypes and time points for parameters showing significant differences were conducted using the least significant difference test via the emmeans package (version 1.10.1; Lenth, 2025). Statistical differences in the number of newly formed root tips between rootstocks at each time point in the side experiment were assessed using a one-way ANOVA, implemented in base R. For allometric analyses, standardized major axis (SMA) regression was performed on log–log transformed data to evaluate the relationship between above- and below-ground parameters of each genotype separately. Analyses were conducted using the smatr package (version 3.4-8; Warton et al., 2012) with default function parameters. We employed partial least squares discriminant analysis (PLS-DA) to differentiate grapevine genotypes based on their architectural traits, using the ropls package (version 1.34.0; Thevenot, 2016). Prior to analysis, all predictor variables were standardized to unit variance and the analysis was conducted using the non-linear iterative partial least squares algorithm by default settings of the ropls package. The PLS-DA was performed with two predictive components (predI = 2) and no orthogonal components (orthoI = 0). Soil moisture data were interpolated using a kriging interpolation model (gstat package, version 2.1-3; Gräler et al., 2016) to generate a continuous soil moisture distribution across depths and time. To assess differences in soil moisture variability between soil horizons, a Levene’s test for homogeneity of variance was performed using the car package (version 3.1-3; Fox and Weisberg, 2019).

## RESULTS

### Chemical and physical soil properties

Chemical analyses of soil samples revealed that SOM was 2.2 ± 0.3 %, with no significant differences between the two depth intervals (0–30 and 30–60 cm; *P* > 0.05) or positions (under-vine versus in-row; *P* > 0.05). Organic carbon (SOC) and nitrate nitrogen ($NO_{3}$–N) was significantly (*P* < 0.05) higher in the upper soil layer (0–30 cm: 1.6 ± 0.2 % SOC; 68 ± 9.1 mg kg^−1^$NO_{3}$–N) compared to the lower layer (30–60 cm: 1.0 ± 0.1 % SOC; 19 ± 2.8 mg kg^−1^$NO_{3}$–N), but did not differ by position. Total nitrogen content (0.12 ± 0.02 %) and the C/N ratio (11 ± 0.6) did not differ significantly by depth or position (all *P* > 0.05). For physical soil properties, soil bulk density measured at 30, 60 and 90 cm in samples from under-vine and under the tractor traffic lane ranged from 1.3 to 1.5 g cm^−3^; although no significant effects of depth or position were detected (both *P* > 0.05), a significant block effect (*P* < 0.01) was found.

### Weather and soil moisture conditions

The 2023 growing season (April–October) was notably warmer (mean temperature of 17 °C) and slightly wetter (334 mm) than the 1991–2020 long-term average (15.7 °C and 323 mm; Supplementary Data Fig. S1). Monthly mean temperatures consistently exceeded the reference period, apart from April. Although total annual precipitation reached 587 mm (about 60 mm above the long-term mean) and the annual mean temperature was 12.4 °C (1.4 °C above the reference), May and June remained exceptionally dry, recording only 27 mm (54 % of the long-term mean) and 5 mm (10 %), respectively. The experimental year 2024 was also warmer than the historical norm (12.2 °C versus 11.0 °C) and received even higher precipitation (660 mm, +25.3 % above the 1991–2020 reference). During the 2024 growing season (16.5 °C mean temperature), a total of 453 mm was recorded (130 mm above average), with May, June and July surpassing their long-term means by 102 %, 42 %, and 52 %, respectively.

Soil moisture measurements reflected these precipitation patterns, showing marked fluctuations in the upper soil layers (0–60 cm) and more stable moisture levels below 60 cm throughout both seasons. A Levene’s test for homogeneity of variance revealed a highly significant difference in soil moisture variability between the two soil horizons (*P* < 0.001), indicating that the upper soil horizon experienced considerably greater fluctuations than the deeper layers and that subsoil moisture was maintained throughout the experiment.

### Early root and shoot development after planting

**Fig. 1.**
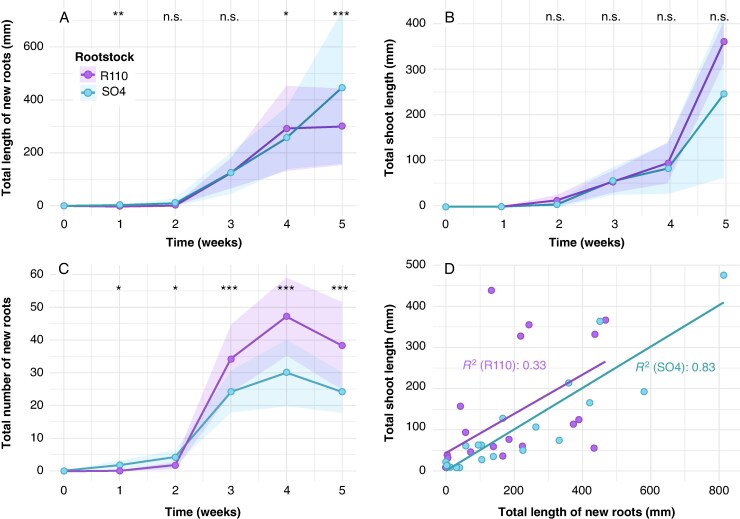
Early root and shoot development of grafted grapevines under field conditions over five weeks after plantation. (A) Total length of new roots (mm), (B) total shoot length (mm) and (C) total number of new roots observed for SO4 (light blue) and R110 (purple) rootstocks across five time points (0–5 weeks). Each point corresponds to the mean of five grapevines excavated per rootstock per time point (*n* = 5). The shaded areas represent the standard deviation. Significant differences between rootstocks were assessed by ANOVA at each time point and are indicated by asterisks: *P < 0.05, **P < 0.01, ***P < 0.001, n.s. (non-significant). (D) Correlation between total shoot length and total length of new roots for all data points across five excavation campaigns (*n* = 50). Each point corresponds to an individual grapevine.

Root initiation was observed one week after planting in SO4 and two weeks in R110, with total root length (Fig. 1A) increasing sharply between weeks 2 and 3 for both genotypes. By week 5, SO4 exhibited significantly longer roots (*P* < 0.001), despite having fewer new roots (Fig. 1C) than R110 (*P* < 0.05). This pattern suggests that R110 invests in a larger number of shorter roots, whereas SO4 develops fewer but longer roots. Shoot growth (Fig. 1B) also increased steadily in both rootstocks, although R110 showed marginally longer shoots by week 5 (*P* > 0.05). The correlation between shoot and root growth (Fig. 1D) was substantially stronger in SO4 (*R*^2^ = 0.83) compared to R110 (*R*^2^ = 0.33), indicating that SO4’s root elongation is more tightly coupled to shoot extension. Conversely, R110 generated more, shorter roots without a proportional increase in shoot length.

### Biomass accumulation and partitioning

**Fig. 2.**
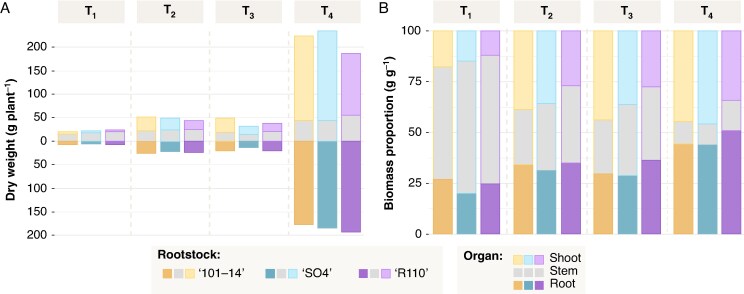
Total grapevine biomass (A) and corresponding biomass proportions (B) of the three grapevine rootstocks (101-14, SO4 and R110) across the four time points (T1–T4). In (A), each stacked bar represents the mean dry weight (g plant^−1^) partitioned into shoot, stem and root. (B) shows the relative contribution of each organ compartment to the total woody biomass at each time point. Additional numerical results and statistical analyses are provided in Supplementary Data Table S2.

Figure 2A illustrates the progressive increase in absolute dry weight for woody plant organs (shoots, stems and roots) across the four time points (T1–T4), whereas Fig. 2B shows the corresponding shifts in biomass proportions. Overall, total vine biomass rose substantially over time (*P* < 0.001), with T4 exhibiting the highest values in all organ compartments. Leaf dry weight (Supplementary Data Table S2) differed significantly among rootstocks (*P* < 0.001), with 101-14 showing higher leaf biomass at T3 than both SO4 and R110 (*P* < 0.001), whereas shoot (*P* > 0.05) and root (*P* > 0.05) dry weights did not vary notably among genotypes. Stem biomass was significantly influenced by rootstock (*P* < 0.001): R110 consistently displayed higher stem dry weights than 101-14 and SO4 at all time points (*P* < 0.001).

Regarding biomass partitioning, shoot woody mass fraction differed significantly by rootstock (*P* < 0.001) and increased markedly over time (T4 > T3 > T2 > T1). In contrast, the stem woody fraction was also rootstock dependent (*P* < 0.001), with SO4 and R110 exhibiting higher stem fractions at T1 than 101-14, but converging by T4. For the root woody mass fraction (*P* < 0.001), R110 attained the highest values at T4 (approximately 0.51 ± 0.003), whereas 101-14 and SO4 remained slightly lower (approximately 0.44 and 0.45, respectively). Although block effects were detected for certain parameters (e.g. shoot woody mass fraction, *P* < 0.001), they did not substantially alter the overall trends, confirming that time and genotype are the primary drivers of biomass accumulation and partitioning. Detailed numerical results and statistical analyses for each parameter are provided in Supplementary Data Table S2.

### Shoot and leaf architecture

**Fig. 3.**
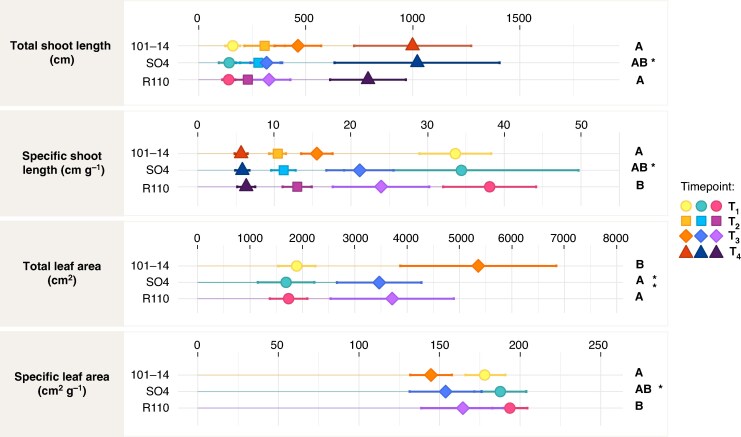
Shoot architecture parameters for three grapevine rootstocks (101-14, SO4 and R110) measured at the four time points (T1–T4). Each panel displays the mean value (symbol) and standard deviation (error bar) of one parameter across time: total shoot length (cm), specific shoot length (cm g^–1^), total leaf area (cm^2^) and specific leaf area (cm^2^ g^–1^). Symbols denote the different time points, with colours corresponding to each rootstock, and significance letters or asterisks indicate statistically significant differences among genotypes: *P < 0.05, **P < 0.01, ***P < 0.001, n.s. (non-significant). Detailed numerical results and statistical analyses are available in Supplementary Data Table S3.

As illustrated in Fig. 3, total shoot length (including main and secondary shoots) increased significantly over time (T1–T4; *P* < 0.001), and differed among rootstocks (*P* < 0.05), with SO4 reaching the greatest length by T4 (1020.4 cm), followed by 101-14 (1000.4 cm) and R110 (791.8 cm). This pattern was also reflected in the main shoot length (*P* < 0.01), where 101-14 maintained higher values than R110 at most time points (Supplementary Data Table S3). In contrast, R110 produced fewer secondary shoots (*P* < 0.001) but did not significantly differ in secondary shoot length (*P* > 0.05). The total number of phytomers likewise showed a small but significant rootstock effect (*P* = 0.014), whereas internode lengths varied primarily over time, with non-significant differences among genotypes. Specific shoot length significantly differed among rootstocks (*P* < 0.005), with R110 exhibiting the highest values overall, whereas time had a significant effect (*P* < 0.001), with lowest values observed at T4.

Leaf area parameters also exhibited strong temporal trends (*P* < 0.001), with all genotypes achieving markedly higher total leaf areas at T3 than at T1. Total leaf area differed significantly by rootstock (*P* < 0.01), and 101-14 had the highest mean values at T3 (5354.1 cm^2^). Moreover, 101-14 displayed significantly larger main shoot leaves (*P* < 0.001) than both SO4 and R110 throughout the experiment. Although mean secondary leaf size did not differ among rootstocks (*P* > 0.05), specific leaf area (*P* < 0.05) was highest in R110 and lowest in 101-14, and decreased with vine age (T3 < T1).

### Root system architecture

#### Global root system architecture

**Fig. 4.**
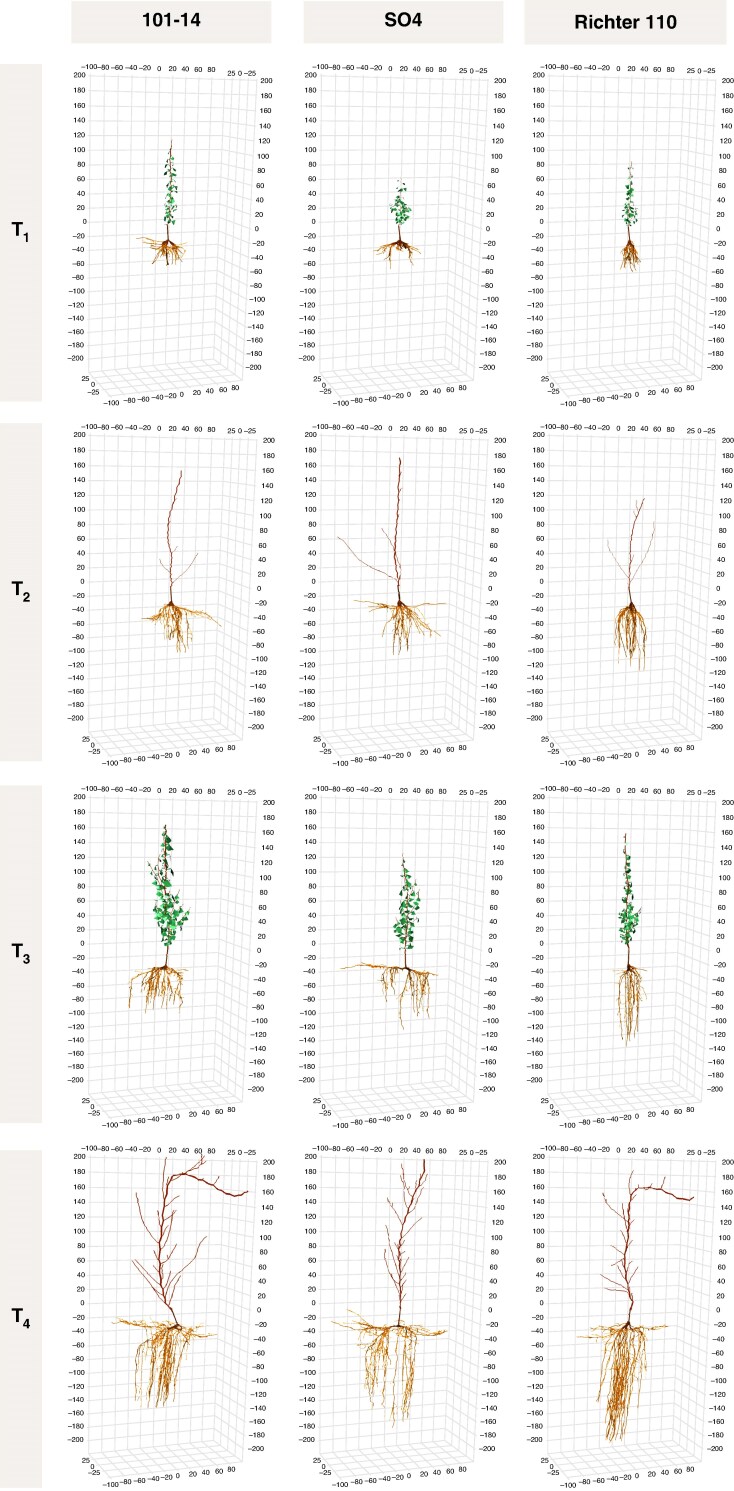
Exemplary 3-D reconstructions of grapevines from each rootstock genotype (101-14, SO4, R110) and time point (T1–T4). Columns correspond to genotypes and rows depict progressive plant development over time. The coordinate axes are in centimetres, and the origin (0,0,0) represents the grafting point, which is approximately located 5 cm above the soil surface.

**Fig. 5.**
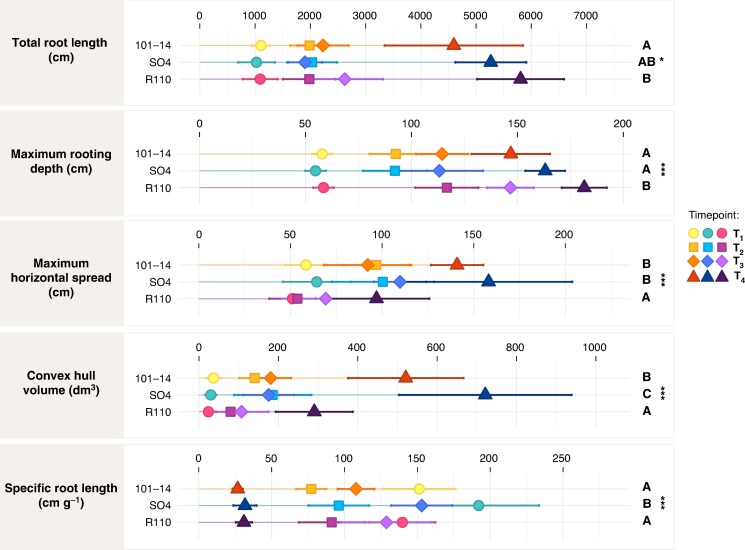
Root system architecture parameters for three grapevine rootstocks (101-14, SO4 and R110) measured at the four time points (T1–T4). Each panel displays the mean value (symbol) and standard deviation (error bar) of one parameter across time: total root length (cm), maximum rooting depth (cm), maximum horizontal spread (cm), convex hull volume (dm^3^), specific root length (cm g^−1^). Symbols denote the different time points, with colours corresponding to each rootstock, and significance letters or asterisks indicate statistically significant differences among genotypes: *P < 0.05, **P < 0.01, ***P < 0.001, n.s. (non-significant). Detailed numerical results and statistical analyses are available in Supplementary Data Table S4.

Representative 3-D reconstructions of each rootstock at T1–T4 (Fig. 4) illustrate the progressive expansion of the root system, both vertically and laterally. Across all genotypes, total root length increased significantly over time (*P* < 0.001), with R110 ultimately reaching the greatest length at T4 (5806 cm), followed by SO4 (5267 cm) and 101-14 (4597 cm; Supplementary Data Table S4 and Fig. 5). A similar trend was observed for maximum rooting depth, where R110 exhibited consistently deeper root penetration (*P* < 0.001), surpassing both 101-14 and SO4 by T4 (181.6 cm versus 146.9 cm and 163.2 cm, respectively). In contrast, 101-14 and SO4 achieved larger horizontal spreads (*P* < 0.001), exceeding 140 cm and 158 cm at T4, respectively, whereas R110 remained at 96.9 cm.

The convex hull volume, reflecting the overall 3-D spatial extent of the root system, also increased markedly with time (*P* < 0.001). By T4, SO4 occupied the largest volume (721.1 dm^3^), while 101-14 reached 520.8 dm^3^ and R110 290.5 dm^3^ (*P* < 0.001). SRL, defined as root length per unit of root dry weight, displayed pronounced differences across both genotype and time (*P* < 0.001). Early in the season (T1), SO4 maintained the highest SRL (192.1 cm g^−1^), whereas by T4, all rootstocks converged to lower values (approximately 27–32 cm g^−1^). Although a significant rootstock × time interaction was detected for total root length (*P* < 0.05) and maximum rooting depth (*P* < 0.001), no significant block effects emerged for these global RSA parameters. Detailed numerical values and statistical analyses are provided in Supplementary Data Table S4.

#### Root length distribution by soil depth

**Fig. 6.**
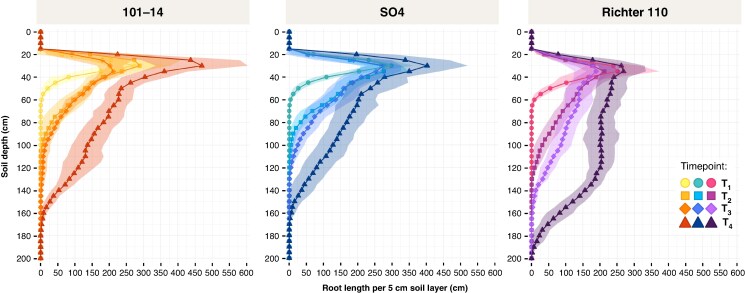
Root length distribution by soil depth for three grapevine rootstocks (101-14, SO4 and R110) at four time points (T1–T4). Each panel corresponds to a specific rootstock, with soil depth (cm) shown on the vertical axis and the total root length (cm) within each 5-cm layer on the horizontal axis. Symbols indicate time points; coloured lines connect the mean values at each time point; shaded areas denote standard deviation. Additional numerical values and statistical analyses on root lengths per soil horizon are provided in Supplementary Data Table S5.

Root length distribution across soil depths increased markedly over time for all genotypes (Fig. 6; *P* < 0.001), with a notable shift towards deeper layers as the vines aged. In the uppermost soil horizon (0–30 cm), root length differed significantly among rootstocks (*P* < 0.001), with 101-14 and SO4 generally showing greater values than R110, particularly at T4, where SO4 reached the highest root length (1177.6 ± 314.7 cm, group C), followed by 101-14 (915.1 ± 342.2 cm, group B), while R110 remained significantly lower (445.5 ± 178.8 cm, group A). However, in deeper layers (e.g. 91–120 cm and beyond), R110 showed the most pronounced increase over time, exceeding 1200 cm by T4 and surpassing both 101-14 and SO4 (*P* < 0.001). Although some depth intervals (31–60 cm) did not display a significant rootstock effect (*P* > 0.05), others (e.g. 61–90 cm, 91–120 cm) revealed strong genotype- and time-dependent trends. Between T2 and T3, a significant increase of root length was only observed in the 61–90 cm soil layer (*P* < 0.05), whereas root length in all other soil depths remained statistically stable for all genotypes at that time step. Significant rootstock × time interactions (e.g. at 91–120 cm, *P* < 0.001) underscore that R110 accelerated its downwards exploration more rapidly at later time points. Detailed numerical results and statistical analyses for each soil horizon are provided in Supplementary Data Table S5.

#### Horizontal root distribution and cropping implications

**Fig. 7.**
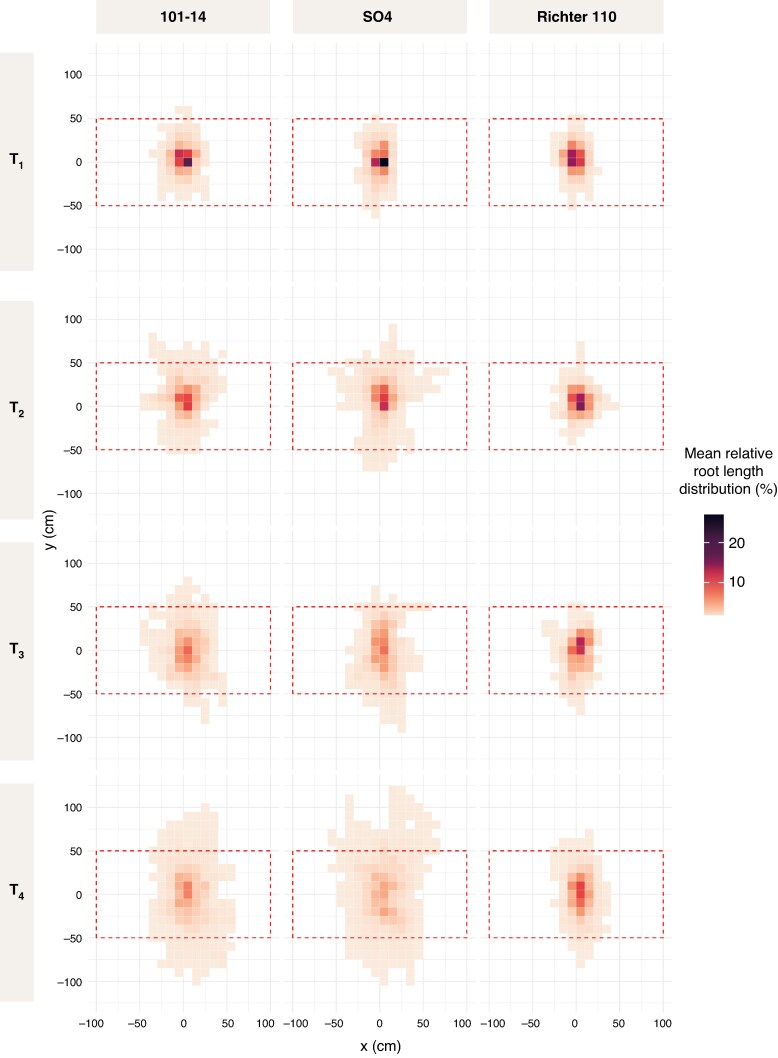
Mean relative root length distribution from a top view across different grapevine rootstocks (columns: 101-14, SO4 and R110) at four time points (rows: T1–T4). Root distribution is shown in relation to X, which represents the direction perpendicular to the vine row, and Y, which represents the direction parallel to the vine row. Each heatmap displays the relative spatial distribution of root length, averaged over eight excavated vines per rootstock at each time point. Root density was calculated by binning spatial coordinates into equal-sized grid cells, followed by normalization to express the relative percentage of root occurrence within each bin. The red dashed box indicates the theoretically available soil area per vine, based on the 2 × 1 m vine planting distance. Darker colours indicate areas with higher relative root density, whereas lighter colours indicate regions with lower root presence. Additional numerical values and statistical analyses on RSA parameters on the XY plane are provided in Supplementary Data Table S6.

Top-down density plots (Fig. 7) reveal that root systems expanded laterally with vine age, although the extent of this spread varied among genotypes. The convex area (Supplementary Data Table S6) increased significantly over time (*P* < 0.001) and differed markedly by rootstock (*P* < 0.001), with SO4 reaching the largest mean area at T4 (90.8 dm^2^), followed by 101-14 (69.9 dm^2^), whereas R110 remained comparatively constrained (31.4 dm^2^). Although the aspect ratio showed a significant time effect (*P* < 0.05), it did not vary among genotypes (*P* > 0.05), suggesting that all rootstocks increasingly expanded in the planting-row direction over time, without pronounced rootstock-specific differences in horizontal dimensions. In contrast, the proportion of roots extending beyond the designated planting area was strongly rootstock-dependent (*P* < 0.001): by T4, SO4 and 101-14 had 10.0 % and 8.3 % of their root length extending into adjacent vine spaces, respectively, whereas R110 barely exceeded 0.6 %. The DBI remained negative for all genotypes, reflecting a stronger orientation along the planting row rather than the inter-row space; however, this bias shifted significantly over time (*P* < 0.001) but did not differ among rootstocks (*P* > 0.05).

### Root–shoot allometry

**Fig. 8.**
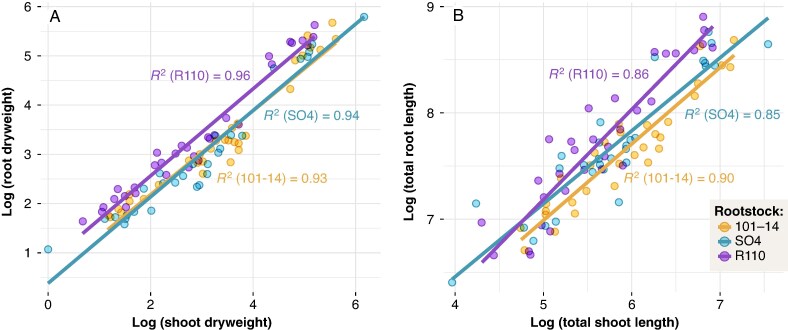
Allometric relationships between root and shoot compartments in grapevines grafted onto three rootstocks (101-14, SO4 and R110). (A) plots the log-transformed root dry weight against the log-transformed shoot dry weight, whereas (B) shows the log-transformed total root length versus the log-transformed total shoot length. Each point represents an individual vine, colour-coded by rootstock. Regression lines, derived from standardized major axis (SMA) models, and corresponding *R*^2^ values illustrate genotype-specific differences in allometric scaling. Additional numerical results and statistical analyses on root-to-shoot ratios are provided in Supplementary Data Table S7.

As depicted in Fig. 8, R110 consistently exhibited the highest root–shoot biomass ratio (e.g. 2.1 ± 0.4 at T1, 1.5 ± 0.3 at T4), whereas 101-14 and SO4 generally showed lower values (Supplementary Data Table S7). This rootstock effect was highly significant (*P* < 0.001), and all genotypes displayed a strong temporal response (*P* < 0.001), with the ratio declining after T1. A similar trend emerged for the root–shoot length ratio (*P* < 0.001): although both 101-14 and SO4 decreased to around 4.6–5.7 by T4, R110 maintained a higher ratio (7.6 ± 1.5).

SMA regressions (Fig. 8, panels A,B) further underscore these differences. For biomass, the slopes (0.89–0.91) were comparable among genotypes, but R110 featured a higher intercept (0.73) than 101-14 (0.37) or SO4 (0.32), indicating a proportionally greater root investment when shoot mass is low. In the length-based SMA models, R110 had a steeper slope (0.92) than 101-14 (0.76) and SO4 (0.75), suggesting that root elongation in R110 scales more modestly with shoot extension relative to the other genotypes. All models showed high coefficients of determination (*R*^2^ > 0.84), demonstrating a robust allometric relationship between above- and below-ground growth. Additional numerical results and statistical details are presented in Supplementary Data Table S7.

### Whole-plant architectural assessment via PLS-DA

**Fig. 9.**
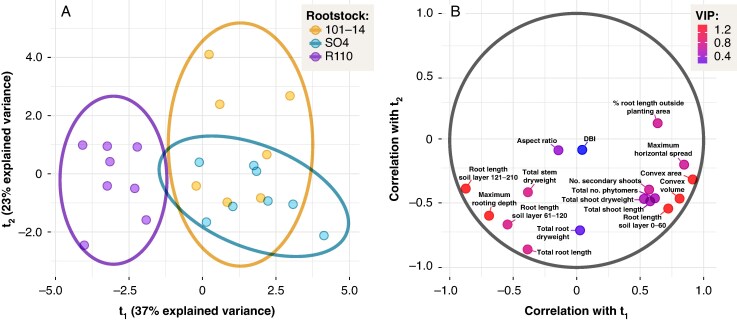
Partial least squares discriminant analysis (PLS-DA) of morphological and architectural parameters for three grapevine rootstocks (101-14, SO4 and R110) at T4. (A) Score plot of the first two latent variables (*t*_1_ and *t*_2_), with points representing individual vines colour-coded by rootstock; ellipses depict 95 % confidence regions. (B) Correlation/loading plot showing the relationship of each parameter to *t*_1_ and *t*_2_, where point colour denotes variable importance in projection (VIP) values. Additional numerical results on VIP scores and loadings are provided in Supplementary Data Figure S8.

To explore overall phenotypic differences at T4, a PLS-DA was conducted using 17 morphological and architectural parameters (Fig. 9, panels A,B). The resulting two-component model explained 59.7 % of the predictor variance ($R^{2}X_{\mathrm{cum}}$) and 52.2 % of the response variance ($R^{2}Y_{\mathrm{cum}}$), with a cross-validated $Q_{\mathrm{cum}}^{2}$ of 0.241 (*P* + 0.05). Although the separation among genotypes was modest, 101-14 tended to cluster along positive $t_{1}$ scores, SO4 showed intermediate scores, and R110 clustered more negatively on $t_{1}$. Variables contributing most to group separation (VIP > 1) included root system dimensions in deep soil layers (root length in soil layer 121-210), maximum rooting depth, and convex area on the XY plane. The loading plots (Supplementary Data Fig. S8) further indicate that total root length, total root dry weight and root length in soil layer 61–120 had strong negative loadings on $t_{2}$, distinguishing R110 from the other genotypes. Overall, the PLS-DA suggests that root system exploration at deeper layers and the horizontal spread of root systems were key discriminators of genotype-specific architecture at the end of the second growing season.

## DISCUSSION

The utilization of rootstock genotypes adapted to specific growing conditions is widely recognized as a key strategy to enhance resource-use efficiency and resilience to environmental stress in grafted perennial crop plants, in particular grapevines (Fraga et al., 2012; Van Leeuwen and Darriet, 2016; Simonneau et al., 2017). Although rootstocks affect scion vigour and water use through mechanisms such as hydraulic conductance and hormonal signalling (Soar et al., 2006; Alsina et al., 2011; Marguerit et al., 2012), the role of root system architectural traits in these responses remains less well understood (Maeght et al., 2013; Zhang et al., 2016). To address this gap, our study used high-resolution 3-D phenotyping to investigate how rootstock genotype shapes whole-plant architecture during early vineyard establishment.

### Deep rooting and biomass allocation patterns reflect strong genetic control

Our findings complement earlier greenhouse studies (Fort et al., 2017; Cuneo et al., 2021) by demonstrating that rootstock-specific differences in both root and shoot architecture also emerge clearly under field conditions. These findings also extend previous work by overcoming the spatial and developmental limitations of pot experiments, which typically constrain root system architecture (RSA) and do not fully capture the full extent and complexity of perennial root systems (Fichtl et al., 2024). A key insight from our field study was the observation of distinct architectural patterns across genotypes even during comparably wet seasons, underscoring that such traits are not merely plastic responses to transient environmental conditions but reflect constitutive traits rather than drought responses. This is supported by the fact that even with sufficient plant-available water in the topsoil layer, deep rooting was observed across rootstocks, highlighting how genetic predisposition can override short-term environmental signals (Blois et al., 2023) under field conditions. Notably, 110R consistently exhibited pronounced vertical rooting, with roots extending beyond 1.80 m within the first two years, a deep-rooting trait that aligns with drought-tolerant ideotypes (Lynch, 2013). These findings are consistent with earlier reports suggesting that RSA in grapevine is largely under genetic control (Southey and Archer, 1988; Soar et al., 2006), provided that soil penetration is not physically constrained, for instance by root-impenetrable layers, anoxia or shallow bedrock (Smart et al., 2006). Such rooting depths are rarely documented empirically, not because they are biologically implausible, but because deep portions of the root system remain understudied. Most investigations of RSA in perennial crops terminate at depths of 1.0–1.2 m due to practical limitations such as shallow coring techniques, restricted trenching depth, or an observational bias towards the topsoil (de Herralde et al., 2010). As a result, deep rooting capacity may be systematically underestimated, although historical reports suggest that grapevine roots can reach depths greater than 6 m under favourable conditions (Seguin, 1972). Given that our young vines reached nearly 2 m depth within 2 years, these earlier accounts appear plausible, at least under deep, well-structured soils.

In terms of biomass allocation, our allometric analyses of shoot and root biomass support functional equilibrium theory, as both scaled proportionally and SMA slopes did not differ substantially from unity. However, genotype-specific shifts in intercepts indicate a genetically tuned allocation strategy, aligning with findings from Tandonnet et al. (2018), who demonstrated a largely independent genetic control of root and aerial traits in graft combinations. Notably, 110R displayed a clear shift toward below-ground allocation at the end of the experiment (18 months after planting), with 51 % of biomass allocated to roots and 34 % to shoots, whereas the other genotypes maintained nearly balanced allocation patterns. This stronger below-ground investment likely reflects increased root sink strength, which has been proposed as a key trait for drought adaptation in woody perennials (Marcelis, 1996). This prioritization of root growth may enhance water and nutrient acquisition and promote resilience under stress conditions. Furthermore, the observed allocation strategies may also reflect differing environmental adaptations rooted in the genotypes’ breeding histories. 110R was selected in the Mediterranean climate of southern France and shows traits consistent with adaptation to water-limited environments, such as deep rooting and enhanced root investment while limiting transpirational demand via reduced shoot and leaf expansion. In contrast, 101-14 and SO4, bred in more temperate regions (France and Germany, respectively), exhibited greater shoot investment and larger leaf area, as evidenced by our data, both of which are traits potentially advantageous in light-limited environments.

We also observed a distinct temporal pattern in biomass allocation, in particular between T2 and T3 (November–July): total biomass accumulation plateaued and root dry mass even slightly declined. This may reflect root dieback during dormancy or root turnover without net increases in total root length or biomass – both processes that remain poorly characterized in perennial fruit crops (Buwalda, 1993). A marked increase in biomass occurred at T4, with root mass increasing up to 10-fold relative to T3. Despite shoot pruning after the first season, shoot biomass at T4 matched root biomass, suggesting that above-ground growth is either prioritized or more rapidly restored following pruning, consistent with optimal allocation theory (Bloom et al., 1985; Freschet et al., 2018). Our investigations also demonstrated temporal root and shoot growth coordination: bud break consistently coincided with initial root emergence, supporting a model of synchronized phenology (Bloom et al., 1985; Tandonnet et al., 2010; Tardieu, 2012). However, studies in other contexts have reported asynchronous shoot and root development in grapevine (Radville et al., 2016), indicating that coordination may depend on genotype, environment or developmental stage.

Taken together, these genotype-specific patterns emphasize the rootstock’s role in regulating the balance between above-ground vegetative growth and below-ground resource acquisition – traits central to long-term performance in grapevine. However, to assess the persistence and agronomic relevance of these allocation patterns beyond the establishment phase, complementary data from mature vineyards are essential.

### 3-D whole-plant architecture data provides valuable input for dynamic and integrative plant modelling

Our findings reinforce the notion that RSA deserves greater attention in ideotype design and functional modelling under climate stress. Integrating genotype-specific parameters into functional–structural plant models will improve predictions of water uptake, carbon allocation and stress responses under future climate conditions (Meunier et al., 2017; Ndour et al., 2017; Fichtl et al., 2023; Schnepf et al., 2023). Our data set offers direct input for such models, including parameters such as elongation rates, branching patterns and root angles. Previous studies have shown that RSA data can be combined with root hydraulic properties to derive spatially resolved standard uptake fractions, which can further elucidate rootstock-specific differences in potential water uptake (Fichtl et al., 2024). However, a limitation of static 3-D data is that they provide snapshots rather than dynamic representations of root growth and turnover. Although our root length distributions offer valuable insights into potential water uptake zones within the soil matrix, the full predictive power of such high-resolution architectural data is realized when integrated into dynamic, process-based growth models. Therefore, future work should incorporate additional time-resolved imaging and modelling approaches to capture dynamic root–soil interactions in heterogeneous field environments.

Recent studies emphasize the need to couple architectural models with water balance simulations to assess vineyard performance under drought (Fichtl et al., 2023). Given strong genotype × environment × management interactions, integrative modelling approaches will be essential to predict optimal rootstock performance under climate change scenarios and parameterized root growth models can help guide rootstock choice for specific soil and climate conditions. Indeed, ideotypes may not be defined by fixed traits, but rather by their plasticity in allocating growth to zones of greatest resource availability (Paez-Garcia et al., 2015). Although this study focused on structural traits, rootstock-mediated physiological processes – such as abscisic acid and cytokinin signalling, or aquaporin regulation – also influence shoot growth (Gambetta et al., 2012; Marguerit et al., 2012; Peccoux et al., 2018). Integrating these signalling mechanisms with architectural data will be crucial for developing computational models of whole-plant performance.

### RSA traits offer practical guidelines for rootstock choice and vineyard management

From a viticultural perspective, genotype-specific RSA traits offer actionable guidance. In drought-prone regions or on sites where deep soil layers are the primary reservoir of plant-available water, deep-rooted genotypes like 110R are advantageous due to their capacity to sustain gas exchange and carbohydrate reserves under stress (Paranychianakis et al., 2004; Fichtl et al., 2023). However, deep rooting alone may not fully explain drought resilience, with additional traits also being critical for maximizing below-ground resource acquisition. These include strong root sink strength, which facilitates preferential assimilate allocation to roots and enhances water acquisition (Postma et al., 2014); steep and cheap root architectures that enable efficient vertical exploration at low carbon cost (Lynch, 2013); and the ability to maintain root growth under declining soil moisture conditions (Bauerle et al., 2008). Although no yield or fruit composition data are available from our study – because young vines do not yet produce fruit – the observed genotype-specific differences in biomass allocation and root-to-shoot ratios are likely to influence future reproductive development and fruit quality, particularly under water-limited conditions. Previous research has shown that below-ground sink strength can have lasting effects on vine performance, including carbohydrate storage and fruit yield potential (Holzapfel et al., 2023). In this context, 110R’s strong root investment and potentially higher root sink strength may enhance resilience and yield stability under drought conditions, even if this does not necessarily translate into higher fruit yield under non-limiting environments. In contrast, such deep-rooting trait may not confer an advantage in vineyard soils with shallow bedrock or limited deep water reserves, where horizontal root expansion and efficient topsoil foraging could prove more beneficial, for example for the acquisition of immobile resources (Tardieu et al., 2017; Lynch, 2019).

However, more shoot-dominant strategies (as observed in 101-14 and SO4) may support rapid canopy development and higher productive potential in resource-rich settings. For instance, in well-irrigated vineyards or high-yielding production systems, rootstocks that favour above-ground allocation and exhibit low root-to-shoot ratios may be beneficial. Such genotypes can promote canopy development and fruiting capacity while maintaining sufficient hydraulic support from a comparatively smaller but efficient root system. In well-resourced environments, such reduced root investment may be advantageous by lowering carbon costs for root construction and maintenance (Buwalda, 1993; Nielsen et al., 1994), allowing more assimilates to be allocated to above-ground growth and reproduction. These contrasting architectural strategies underscore the importance of matching rootstock choice not only to environmental constraints, but also to management regimes and production goals.

The three genotypes also differed in the volume and pattern of soil exploration. Analyses of the root system convex hull volume at T4 showed that SO4 explored the largest soil volume, followed by 101-14 and then 110R. These differences underline the importance of root system architecture not only in vertical depth but also in horizontal spread – traits that can be leveraged to tailor vineyard design and irrigation strategies. Indeed, irrigation can be optimized according to RSA: deep-rooted vines such as 110R might benefit from infrequent, high-volume irrigation, whereas shallow-rooted types may respond better to frequent, low-volume inputs. Our spatial RSA data provide a useful foundation to inform such precision strategies.

Interestingly, root growth was consistently oriented along the vine row independent of genotype, with limited extension into the inter-row space. This aligns with findings by Celette et al. (2008) and may be influenced by mechanical planting practices or subtle compaction gradients. Although bulk density differences were not statistically significant in our study, previous work in similar conditions has shown that traffic lanes can restrict rooting due to increased compaction (Hendgen et al., 2020). This spatial pattern has practical implications: early vineyard stages are often managed without cover crops due to concerns over water competition (Fichtl et al., 2023). However, our data suggest these concerns may be overstated, and that early cover cropping could be employed to improve soil structure and biodiversity without significantly impacting root development.

## CONCLUSION

This study provides one of the most comprehensive field-based assessments of early grapevine architecture to date, integrating detailed 3-D phenotyping of root and shoot traits across genotypes and time points. Our results clearly show that rootstock genotype is a primary determinant of whole-plant architecture, shaping not only rooting depth and verticality but also shoot growth, leaf area and biomass allocation. The deep, vertically biased rooting of drought-tolerant 110R exemplifies a genetically encoded habit for subsoil resource capture and resilience, whereas the more horizontally expansive systems of 101-14 and SO4 reflect strategies optimized for topsoil exploitation under wetter or more fertile conditions. These contrasting patterns underscore the importance of root system architecture as a central component of genotype-specific plant function.

Despite pronounced genotypic differences, all rootstocks displayed coordinated scaling of root and shoot biomass consistent with the functional equilibrium theory. The persistence of architectural traits under non-drought field conditions supports the interpretation that RSA represents a robust, genetically regulated feature, expressed independently of transient environmental stress. Furthermore, our spatial data demonstrate that young grapevine roots predominantly occupy the planting row, challenging assumptions about early inter-row competition with cover crops and opening new opportunities for sustainable groundcover management during vineyard establishment.

From an applied perspective, our findings offer actionable guidelines for rootstock choice tailored to specific site conditions: deep-rooted genotypes like 110R are well-suited to drought-prone environments, whereas more shallow-rooted types may help control excessive vegetative growth in fertile or humid regions. In addition, the 3-D architectural traits characterized here – such as rooting depth, convex hull volume and dynamic biomass allocation – provide key parameters for functional–structural plant models and support more targeted irrigation and management strategies that leverage genotype-specific potential.

Looking ahead, future research should extend these analyses to later developmental stages, a wider range of soil types and more pronounced stress conditions, including drought and nutrient limitations. To fully capture the complexity of genotype–environment interactions and their impacts on yield and quality, architectural phenotyping must be coupled with insights into root–soil dynamics and hormonal signalling. Such integrative, mechanistic approaches are key to developing predictive models that support resilient, productive, and sustainable perennial cropping systems.

Together, these findings reinforce the role of rootstock genotype as a central lever in climate-resilient vineyard systems and lay the groundwork for integrative modelling and breeding approaches.

## Supplementary Material

mcaf193_Supplementary_Data

## Data Availability

The data that support the findings of this study (3-D whole-plant architectural data) are openly available in Zenodo at https://doi.org/10.5281/zenodo.15632062.

## SUPPLEMENTARY INFORMATION

Supplementary data are available at *Annals of Botany* online and consist of the following. Figure S1: Temporal variation in weather and soil moisture during vineyard establishment, from May 2023 to November 2024. Table S2: Numerical and statistical results of biomass allocation. Table S3: Numerical and statistical results of shoot architecture characteristics. Table S4: Numerical and statistical results of global root system architecture characteristics. Table S5: Numerical and statistical results of root length distribution across different soil horizons. Table S6: Numerical and statistical results of horizontal root system architecture characteristics. Table S7: Numerical and statistical results of root-to-shoot ratios. Figure S8: VIP scores and loadings of the PLS-DA.
