# Supplementary material for: Rootstock genotype shapes whole-plant 3-D architecture and biomass allocation in field-grown grapevines
Source: Ann Bot. 2025 Sep 1;136(7):1613–29. doi: 10.1093/aob/mcaf193 (PMC12718041; doi:10.1093/aob/mcaf193)
Supplement: mcaf193_Supplementary_Data [file mcaf193_supplementary_data.pdf]

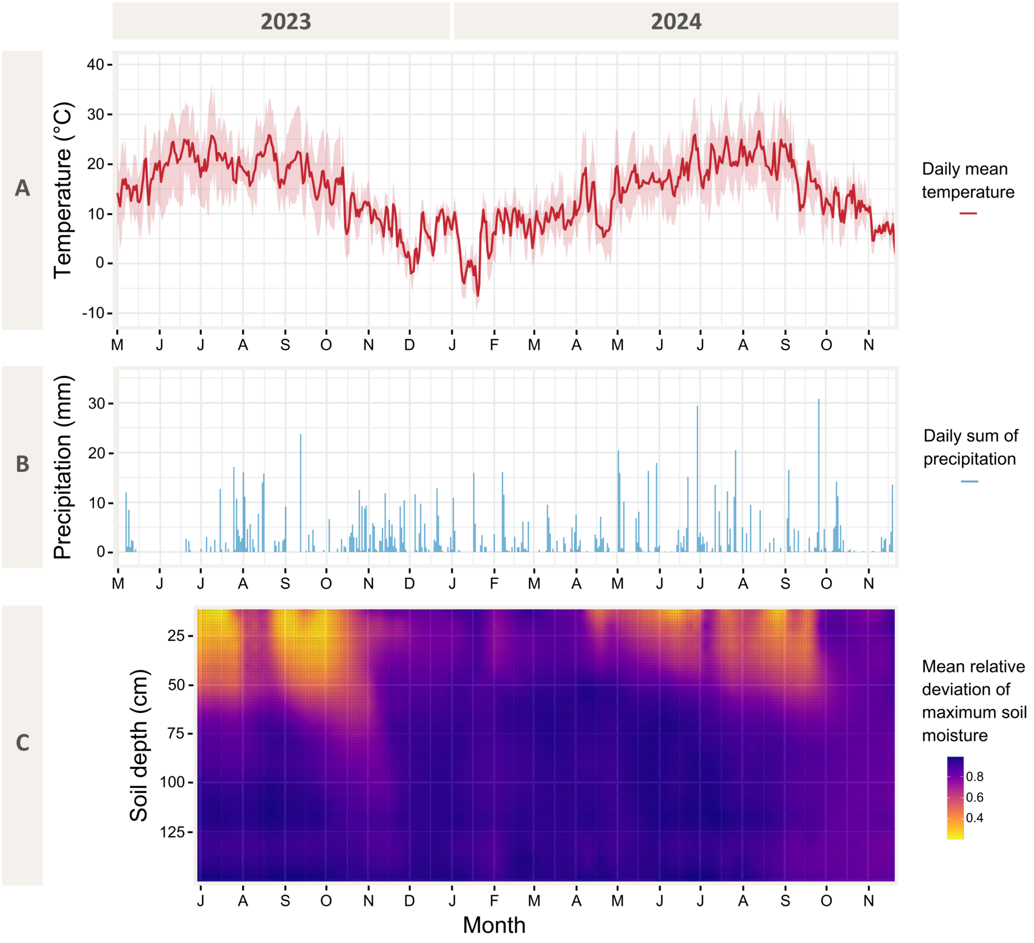

Supplementary Figure S1: Temporal variation in weather and soil moisture during vineyard establishment, from May 2023 to November 2024. (A) Daily mean air temperature at 2 m elevation (°C) recorded at the weather station. The red line represents the daily mean temperature, while the shaded area indicates the range between daily minimum and maximum temperatures. (B) Daily total precipitation (mm) recorded at the weather station, displayed as vertical blue bars. (C) Spatiotemporal distribution of mean relative deviation of maximum soil moisture (estimated as a mean of eight soil moisture access tubes). Warmer colors (yellow) indicate lower soil moisture, while cooler colors (purple) represent higher relative soil moisture availability.

Supplementary Table S2: Summary of biomass accumulation across rootstock genotypes (101-14, SO4, and R110) and timepoints (T1–T4). The table presents mean dry weights (± standard deviation) for leaves, shoots, stems, and roots, as well as total biomass and biomass fractions. Results from linear mixed-effects models are included, showing the effects of rootstock genotype, time, their interaction, and block. Capital letters indicate significant differences among genotypes or timepoints according to post hoc tests (*p* < 0.05). Lowercase letters following numeric values denote significant rootstock × time interactions where present.

**Parameter**	**Rootstock effect**		**Rootstock**	**T1**		**T2**		**T3**		**T4**		**Time effect**	**Interaction rootstock_time**	**Block effect**
Total leaf dry weight (g)				**A**				**B**						
	***	**B**	**101-14**	10.7 ± 2.5	a			37.0 ± 9.4	c			***	*	n.s.
		**A**	**SO4**	9.0 ± 2.6	a			22.7 ± 5.7	b					
	0.0007372	**A**	**R110**	9.0 ± 2.0	a			23.8 ± 11.0	b			7.171e-13	0.0127533	0.2312
Total shoot dry weight (g)				**A**		**A**		**A**		**B**				
	n.s.	**–**	**101-14**	5.0 ± 1.8	–	30.2 ± 10.7	–	30.8 ± 9.8	–	179.6 ± 59.1	–	***	n.s.	n.s.
		**–**	**SO4**	4.1 ± 1.8	–	25.7 ± 11.4	–	15.7 ± 5.5	–	190.7 ± 119.2	–			
	0.1198	**–**	**R110**	3.8 ± 1.3	–	18.3 ± 8.6	–	16.2 ± 11.2	–	129.9 ± 41.2	–	<2e-16	0.3345	0.8858
Total stem dry weight (g)				**A**		**B**		**A**		**C**				
	***	**A**	**101-14**	15.4 ± 3.0	–	20.5 ± 4.2	–	18.5 ± 3.4	–	43.9 ± 6.1	–	***	n.s.	n.s.
		**A**	**SO4**	17.5 ± 1.7	–	23.3 ± 5.4	–	15.2 ± 1.9	–	42.9 ± 9.8	–			
	2.193e-05	**B**	**R110**	20.1 ± 4.3	–	25.2 ± 6.2	–	21.4 ± 3.8	–	55.2 ± 9.0	–	<2.2e-16	0.07411	1
Total root dry weight (g)				**A**		**A**		**A**		**B**				
	n.s.	**–**	**101-14**	7.5 ± 1.8	–	26.4 ± 7.2	–	20.9 ± 4.9	–	178.4 ± 64.6	–	***	n.s.	n.s.
		**–**	**SO4**	5.4 ± 1.1	–	22.2 ± 7.0	–	12.5 ± 2.0	–	183.2 ± 65.0	–			
	0.7480	**–**	**R110**	7.8 ± 1.8	–	23.6 ± 9.8	–	21.4 ± 8.0	–	193.2 ± 7.5	–	<2e-16	0.9842	1
Total woody biomass dryweight (g)				**A**		**A**		**A**		**B**				
	n.s.	**–**	**101-14**	27.8 ± 6.4	–	77.1 ± 20.5	–	70.2 ± 12.5	–	401.8 ± 121.4	–	***	n.s.	n.s.
		**–**	**SO4**	27.1 ± 3.3	–	72.9 ± 19.6	–	43.4 ± 7.2	–	416.9 ± 191.9	–			
	0.8482	**–**	**R110**	31.7 ± 6.7	–	67.1 ± 21.0	–	58.9 ± 19.4	–	378.3 ± 89.3	–	<2e-16	0.9570	1
Shoot woody mass fraction				**A**		**B**		**B**		**C**				
	***	**C**	**101-14**	0.17 ± 0.03	–	0.38 ± 0.05	–	0.43 ± 0.08	–	0.45 ± 0.05	–	***	.	***
		**B**	**SO4**	0.15 ± 0.05	–	0.34 ± 0.11	–	0.35 ± 0.07	–	0.44 ± 0.05	–			
	2.011e-11	**A**	**R110**	0.12 ± 0.03	–	0.26 ± 0.07	–	0.26 ± 0.09	–	0.34 ± 0.05	–	<2.2e-16	0.09541	0.0004823
Stem woody mass fraction				**C**		**B**		**B**		**A**				
	***	**A**	**101-14**	0.56 ± 0.04	–	0.27 ± 0.04	–	0.27 ± 0.08	–	0.11 ± 0.02	–	***	n.s.	n.s.
		**B**	**SO4**	0.65 ± 0.07	–	0.35 ± 0.13	–	0.35 ± 0.05	–	0.11 ± 0.02	–			
	8.553e-07	**B**	**R110**	0.63 ± 0.04	–	0.40 ± 0.10	–	0.38 ± 0.10	–	0.15 ± 0.03	–	<2.2e-16	0.1416	1
Root woody mass fraction				**A**		**B**		**B**		**C**				
	***	**B**	**101-14**	0.27 ± 0.02	b	0.34 ± 0.03	cd	0.30 ± 0.04	bcd	0.44 ± 0.06	e	***	**	n.s.
		**A**	**SO4**	0.20 ± 0.03	a	0.30 ± 0.03	bcd	0.29 ± 0.06	bc	0.45 ± 0.04	ef			
	2.644e-06	**C**	**R110**	0.25 ± 0.02	ab	0.34 ± 0.05	cd	0.36 ± 0.04	d	0.51 ± 0.03	f	<2.2e-16	0.004289	1

Supplementary Table S3: Summary of shoot architectural traits across rootstock genotypes (101-14, SO4, and R110) and timepoints (T1–T4). The table reports mean values (± standard deviation) for parameters including shoot length, number of phytomers, internode length, number and length of secondary shoots, leaf area, and specific leaf traits. Results from linear mixed-effects models are provided for each trait, including the effects of genotype, time, their interaction, and block. Capital letters indicate significant differences among genotypes or timepoints based on post hoc comparisons (p < 0.05). Lowercase letters following numeric values denote significant genotype × time interactions where applicable.

Parameter	Rootstock effect		Rootstock	T1		T2		T3		T4		Time effect	Interaction rootstock_time	Block effect
Total shoot length (cm)	*			A		B		B		C		***	n.s.	n.s.
		B	101-14	159.7 ± 35.0	–	308.0 ± 94.6	–	464.4 ± 108.7	–	1000.4 ± 273.6	–			
	0.02369	AB	SO4	143.9 ± 49.7	–	279.8 ± 104.6	–	317.3 ± 73.9	–	1020.4 ± 385.3	–	< 2e-16	0.28711	0.4339
		A	R110	140.6 ± 30.5	–	226.7 ± 91.9	–	330.1 ± 99.1	–	791.8 ± 176.6	–			
Main shoot length (cm)	**			A		B		B		C		***	n.s.	.
		B	101-14	83.9 ± 28.4	–	189.4 ± 27.5	–	166.0 ± 34.4	–	247.9 ± 37.9	–			
	0.006218	AB	SO4	75.5 ± 29.4	–	166.9 ± 56.6	–	126.9 ± 36.2	–	261.8 ± 50.2	–	< 2-2e-16	0.367195	0.05725
		A	R110	66.3 ± 16.6	–	132.0 ± 43.5	–	130.3 ± 53.3	–	233.4 ± 44.0	–			
Number of secondary shoots (n)	***			B		A		B		C		***	n.s.	n.s.
		B	101-14	12.5 ± 3.4	–	8.1 ± 3.6	–	12.8 ± 1.4	–	19.3 ± 4.9	–			
	0.0007513	B	SO4	11.3 ± 4.1	–	6.0 ± 2.7	–	10.4 ± 2.7	–	20.5 ± 2.7	–	< 2.2e-16	0.5589580	0.2291
		A	R110	10.3 ± 2.8	–	4.4 ± 2.6	–	9.6 ± 3.1	–	16.0 ± 3.6	–			
Secondary shoot length (cm)	.			A		A		B		C		***	n.s.	n.s.
		–	101-14	75.8 ± 19.9	–	118.7 ± 84.8	–	298.5 ± 99.8	–	752.5 ± 254.7	–			
	0.08719	–	SO4	68.4 ± 28.2	–	112.8 ± 71.8	–	190.3 ± 49.3	–	758.6 ± 345.6	–	< 2e-16	0.25712	0.9326
		–	R110	74.3 ± 22.6	–	94.6 ± 64.5	–	199.8 ± 56.0	–	558.4 ± 174.4	–			
Total number of phytomers (n)	*			A		A		A		B		***	n.s.	n.s.
		B	101-14	70.3 ± 13.5	–	80.3 ± 19.4	–	91.4 ± 12.4	–	253.8 ± 69.4	–			
	0.0140	AB	SO4	66.3 ± 18.3	–	69.0 ± 22.4	–	75.8 ± 15.2	–	255.6 ± 72.1	–	< 2e-16	0.2032	0.2799
		A	R110	62.6 ± 13.4	–	63.1 ± 23.6	–	73.6 ± 15.2	–	198.4 ± 35.2	–			
Number of main phytomers (n)	*			A		B		A		B		***	n.s.	.
		B	101-14	23.0 ± 5.7	–	40.3 ± 2.5	–	24.6 ± 3.4	–	38.3 ± 4.8	–			
	0.02515	AB	SO4	22.6 ± 4.8	–	35.4 ± 8.0	–	22.9 ± 1.8	–	37.6 ± 2.7	–	< 2e-16	0.61793	0.09025
		A	R110	20.9 ± 2.9	–	32.9 ± 6.7	–	22.4 ± 4.5	–	36.8 ± 7.2	–			
Number of secondary phytomers (n)	*			AB		A		B		C		***	n.s.	n.s.
		B	101-14	47.5 ± 9.7	–	40.1 ± 19.9		71.0 ± 12.8	–	218.3 ± 67.2	–			
	0.03177	AB	SO4	44.4 ± 14.8	–	34.1 ± 18.2	–	56.0 ± 13.4	–	220.8 ± 72.8	–	< 2e-16	0.16450	0.443
		A	R110	42.3 ± 12.5	–	30.5 ± 19.8	–	54.8 ± 13.0	–	164.6 ± 36.3	–			
Mean main internode length (cm)	.			A		B		C		C		***	n.s.	n.s.
		–	101-14	3.61 ± 0.57	–	4.71 ± 0.63	–	6.71 ± 0.83	–	6.53 ± 1.03	–			
	0.05267	–	SO4	3.24 ± 0.71	–	4.60 ± 0.79	–	5.60 ± 1.71	–	7.00 ± 1.32	–	< 2e-16	0.42952	0.2662
		–	R110	3.14 ± 0.43	–	3.94 ± 0.68	–	5.69 ± 1.54	–	6.40 ± 0.67	–			
Mean secondary internode length (cm)	n.s.			A		B		C		C		***	n.s.	n.s.
		–	101-14	1.60 ± 0.24	–	2.71 ± 0.72	–	4.12 ± 0.82	–	3.42 ± 0.31	–			
	0.6867	–	SO4	1.52 ± 0.24	–	3.05 ± 0.66	–	3.44 ± 0.52	–	3.33 ± 0.43	–	< 2e-16	0.3073	1
		–	R110	1.76 ± 0.28	–	2.86 ± 0.86	–	3.68 ± 0.80	–	3.34 ± 0.45	–			
Specific shoot length (cm g^{-1})	*			D		B		C		A		***	n.s.	n.s.
		A	101-14	33.6 ± 4.7	–	10.4 ± 1.1		15.6 ± 2.0	–	5.7 ± 0.9	–			
	0.01416	AB	SO4	34.4 ± 15.3	–	11.2 ± 1.6		21.2 ± 4.4	–	5.8 ± 0.9	–	< 2e-16	0.49833	0.1051
		B	R110	38.1 ± 6.0	–	13.0 ± 1.9		23.9 ± 6.3	–	6.2 ± 1.2	–			
Total leaf area (cm^2)	**			A				B				***	*	n.s.
		B	101-14	1893.8 ± 354.9	a			5354.1 ± 1490.0	c					
	0.003336	A	SO4	1686.6 ± 536.5	a			3465.3 ± 809.2	b			1.306e-11	0.021306	0.7557
		A	R110	1735.8 ± 358.4	a			3715.3 ± 1176.4	b					
Total main leaf area (cm^2)	**			A				B				***	.	n.s.
		B	101-14	1004.7 ± 291.1	–			2345.0 ± 498.6	–					
	0.001241	A	SO4	826.2 ± 296.8	–			1528.9 ± 442.8	–			8.316e-10	0.059802	1
		A	R110	802.1 ± 195.9	–			1551.6 ± 591.2	–					
Total secondary leaf area (cm^2)	*			A				B				***	*	n.s.
		B	101-14	889.1 ± 224.4	a			3009.2 ± 1095.2	c					
	0.03750	A	SO4	860.5 ± 288.4	a			1936.4 ± 560.3	b			2.469e-10	0.04322	0.8043
		AB	R110	933.7 ± 276.3	a			2163.7 ± 721.7	bc					
Mean main leaf size (cm^2)	***			A				B				***	*	n.s.
		B	101-14	48.5 ± 4.7	a			121.8 ± 20.6	c					
	0.0004313	A	SO4	41.7 ± 7.9	a			83.2 ± 22.8	b			3.884e-14	0.0143770	0.5862
		A	R110	43.7 ± 5.0	a			88.1 ± 23.3	b					
Mean secondary leaf size (cm^2)	n.s.			A				B				***	n.s.	n.s.
		–	101-14	22.2 ± 3.1	–			50.3 ± 9.8	–					
	0.1893	–	SO4	22.0 ± 2.4	–			42.8 ± 7.9	–			1.903e-14	0.3458	0.5516
		–	R110	25.0 ± 4.5	–			47.6 ± 10.8	–					
Specific leaf area ($cm^2 g^{-1}$)	*			B				A				***	n.s.	n.s.
		A	101-14	177.9 ± 12.8	–			144.7 ± 12.9	–					
	0.01714	AB	SO4	187.6 ± 16.2	–			153.8 ± 22.3	–			1.143e-07	0.94112	0.1466
		B	R110	193.6 ± 10.9	–			164.3 ± 25.6	–					

Supplementary Table S4: Summary of root system architectural traits for grapevine rootstocks (101-14, SO4, and R110) across four timepoints (T1–T4). Reported parameters include total root length, rooting depth, horizontal spread, convex hull metrics, root length density across soil layers, and specific root length. Values are presented as means ± standard deviation. Statistical results from linear mixed-effects models are provided, including effects of genotype, time, genotype × time interactions, and block. Capital letters indicate significant differences among genotypes or timepoints based on post hoc tests ($p < 0.05$), while lowercase letters following numeric values denote significant genotype × time interactions when present.

Parameter	Rootstock effect		Rootstock	T1		T2		T3		T4		Time effect	Interaction rootstock_time	Block effect
Total root length (cm)	*			**A**		**B**		**B**		**C**		***	*	n.s.
		**A**	**101-14**	1105.6 ± 159.9	ab	1985.6 ± 351.6	abc	2228.8 ± 463.2	c	4597.4 ± 1251.6	d			
	0.01834	**AB**	**SO4**	1025.1 ± 335.0	a	2032.0 ± 451.9	bc	1902.7 ± 305.9	abc	5266.6 ± 643.8	de	< 2e-16	0.02605	0.3964
		**B**	**R110**	1092.2 ± 317.1	ab	1980.8 ± 474.2	abc	2623.2 ± 692.7	c	5806.1 ± 787.9	e			
Maximum rooting depth (cm)	***			**A**		**B**		**C**		**D**		***	***	*
		**A**	**101-14**	57.9 ± 4.9	a	92.6 ± 12.5	b	114.5 ± 12.3	c	146.9 ± 18.5	d			
	1.016e-11	**A**	**SO4**	54.8 ± 4.9	a	92.2 ± 15.2	b	113.2 ± 20.7	c	163.2 ± 9.4	de	< 2.2e-16	0.000532	0.01567
		**B**	**R110**	58.5 ± 4.9	a	116.7 ± 14.9	c	146.7 ± 11.1	d	181.6 ± 10.6	e			
Maximum horizontal spread (cm)	***			**A**		**B**		**B**		**C**		***	.	n.s.
		**B**	**101-14**	58.5 ± 11.6	–	96.8 ± 17.7	–	92.1 ± 23.8	–	140.9 ± 14.3	–			
	9.891e-10	**B**	**SO4**	64.3 ± 18.5	–	100.4 ± 27.9	–	109.8 ± 14.3	–	158.1 ± 45.7	–	< 2.2e-16	0.07466	0.2084
		**A**	**R110**	51.0 ± 12.6	–	53.7 ± 15.3	–	69.2 ± 25.0	–	96.9 ± 28.9	–			
Convex hull volume (dm^3)	***			**A**		**B**		**B**		**C**		***	***	ns.s
		**B**	**101-14**	36.4 ± 18.4	abc	140.4 ± 39.6	abcd	180.4 ± 51.5	bcd	520.8 ± 145.6	e			
	1.380e-08	**C**	**SO4**	29.7 ± 14.0	ab	185.5 ± 97.8	cd	175.5 ± 64.0	abcd	721.1 ± 217.5	f	< 2.2e-16	2.204e-07	0.4267
		**A**	**R110**	23.6 ± 8.1	a	79.6 ± 36.1	abc	107.0 ± 68.0	abc	290.5 ± 97.1	d			
Specific root length ($cm\ g^{-1}$)	***			**D**		**B**		**C**		**A**		***	**	n.s.
		**A**	**101-14**	151.3 ± 24.9	e	77.2 ± 10.4	b	107.9 ± 12.7	bcd	26.7 ± 3.6	a			
	7.916e-06	**B**	**SO4**	192.1 ± 41.5	f	96.1 ± 20.8	bc	153.0 ± 20.9	e	31.7 ± 8.0	a	< 2.2e-16	0.009172	0.8921
		**A**	**R110**	139.7 ± 22.7	de	91.2 ± 22.6	b	128.8 ± 31.0	cde	31.0 ± 5.6	a			

Supplementary Table S5: Root length distribution across soil depths (in 30 cm increments) for grapevine rootstocks (101-14, SO4, and R110) at four timepoints (T1–T4). Values represent mean total root length per soil layer (cm) ± standard deviation. Statistical outputs from linear mixed-effects models are included, reporting the main effects of genotype, time, their interaction, and block. Capital letters indicate significant differences among genotypes or timepoints ($p < 0.05$), based on post hoc tests. Lowercase letters following numerical values denote significant genotype × time interactions, where applicable.

Total root length per soil horizon (cm)	Rootstock effect		Rootstock	T1		T2		T3		T4		Time effect	Interaction rootstock_time	Block effect
0 – 30	***			A		A		A		B		***	**	n.s.
		B	**101-14**	573.2 ± 83.2	abc	438.5 ± 147.1	ab	460.6 ± 223.7	ab	915.1 ± 342.2	cd			
	7.997e-09	C	**SO4**	587.3 ± 226.5	abc	731.4 ± 371.0	bc	459.3 ± 208.7	ab	1177.6 ± 314.7	d	1.034e-08	0.00636	0.8004
		A	**R110**	326.4 ± 147.4	a	287.4 ± 120.3	a	358.9 ± 148.0	ab	445.5 ± 178.8	ab			
31 – 60	n.s.			A		B		B		C		***	*	n.s.
		–	**101-14**	508.4 ± 120.4	a	1188.4 ± 242.0	cd	1161.8 ± 327.8	cd	1614.6 ± 342.6	e			
	0.18587	–	**SO4**	415.3 ± 188.2	a	982.5 ± 113.2	bc	967.0 ± 111.0	bc	1677.0 ± 227.2	e	< 2e-16	0.02582	1
		–	**R110**	740.9 ± 307.6	ab	1017.7 ± 264.0	bc	958.1 ± 255.7	bc	1452.5 ± 216.6	de			
61 – 90	***			A		B		C		D		***	n.s.	.
		A	**101-14**	1.5 ± 2.6	–	314.1 ± 151,3	–	470.7 ± 205.9	–	1058.5 ± 297.5	–			
	0.000229	A	**SO4**	0.2 ± 0.5	–	279.7 ± 184.2	–	362.7 ± 184.2	–	1153.2 ± 255.4	–	< 2.2e-16	0.107964	0.05689
		B	**R110**	2.2 ± 3.9	–	491.6 ± 148.8	–	703.2 ± 198.0	–	1250.0 ± 231.9	–			
91 – 120	***			A		A		B		C		***	***	*
		A	**101-14**	0.0 ± 0.0	a	21.8 ± 40.2	a	109.8 ± 86.9	a	692.9 ± 280.7	c			
	1.053e-10	A	**SO4**	0.0 ± 0.0	a	17.0 ± 19.9	a	74.0 ± 79.9	a	793.2 ± 288.1	c	< 2.2e-16	2.058e-05	0.02852
		B	**R110**	0.0 ± 0.0	a	155.7 ± 105.1	a	442.6 ± 148.5	b	1215.4 ± 239.2	d			
121 – 150	***			A		A		A		B		***	***	n.s.
		A	**101-14**	0.0 ± 0.0	a	0.0 ± 0.0	a	2.9 ± 4.6	a	271.1 ± 207.2	bc			
	< 2.2e-16	A	**SO4**	0.0 ± 0.0	a	0.0 ± 0.0	a	15.7 ± 38.3	a	402.3 ± 149.5	c	< 2.2e-16	< 2.2e-16	1
		B	**R110**	0.0 ± 0.0	a	6.8 ± 8.2	a	131.4 ± 58.6	ab	1012.4 ± 141.8	d			
151 – 180	***			A		A		A		B		***	***	n.s.
		A	**101-14**	0.0 ± 0.0	a	0.0 ± 0.0	a	0.0 ± 0.0	a	22.8 ± 39.5	a			
	3.177e-11	A	**SO4**	0.0 ± 0.0	a	0.0 ± 0.0	a	0.8 ± 2.2	a	40.8 ± 36.0	a	< 2.2e-16	< 2.2e-16	1
		B	**R110**	0.0 ± 0.0	a	0.0 ± 0.0	a	6.2 ± 12.8	a	379.1 ± 166.0	b			
181 – 210	*			A		A		A		B		**	***	n.s.
		A	**101-14**	0.0 ± 0.0	a	0.0 ± 0.0	a	0.0 ± 0.0	a	0.0 ± 0.0	a			
	0.0160904	A	**SO4**	0.0 ± 0.0	a	0.0 ± 0.0	a	0.0 ± 0.0	a	0.0 ± 0.0	a	0.0068115	0.0007312	1
		B	**R110**	0.0 ± 0.0	a	0.0 ± 0.0	a	0.0 ± 0.0	a	29.8 ± 40.5	b			

Supplementary Table S6: Horizontal root distribution parameters for grapevine rootstocks (101-14, SO4, and R110) across four timepoints (T1–T4). Parameters include convex hull area (dm^2), aspect ratio, Directional Bias Index (DBI), and the proportion of root length outside the designated planting area. Values are presented as means ± standard deviation. Statistical results from linear mixed-effects models indicate main effects of genotype, time, their interaction, and block. Capital letters denote significant differences among genotypes or timepoints (p < 0.05) based on post hoc tests. Lowercase letters following numeric values signify significant genotype × time interactions where applicable.

**Parameter**	**Rootstock effect**	**Rootstock**		**T1**		**T2**		**T3**		**T4**		**Time effect**	**Interaction rootstock_time**	**Block effect**
Convex Area (dm^2)	***			**A**		**B**		**B**		**C**		***	***	n.s.
		**B**	**101-14**	15.9 ± 6.5	abc	35.4 ± 7.0	bcd	37.6 ± 14.6	cd	69.9 ± 16.0	e			
	1.239e-12	**B**	**SO4**	14.7 ± 5.5	ab	46.7 ± 21.9	d	35.4 ± 12.1	bcd	90.8 ± 28.1	e	< 2.2e-16	2.389e-05	0.7975
		**A**	**R110**	10.5 ± 3.4	a	14.2 ± 5.1	ab	15.7 ± 8.5	abc	31.4 ± 11.0	abcd			
Apect-Ratio	n.s.			**B**		**B**		**AB**		**A**		*	n.s.	n.s.
		**–**	**101-14**	0.88 ± 0.50	–	0.67 ± 0.24	–	0.71 ± 0.17	–	0.52 ± 0.08	–			
	0.20821	**–**	**SO4**	0.57 ± 0.14	–	0.72 ± 0.08	–	0.52 ± 0.15	–	0.58 ± 0.17	–	0.01058	0.14342	1
		**–**	**R110**	0.70 ± 0.21	–	0.84 ± 0.22	–	0.63 ± 0.26	–	0.53 ± 0.14	–			
Proportion root length outside planting area	***			**A**		**A**		**A**		**B**		***	*	n.s.
		**B**	**101-14**	0.2 ± 0.5	a	1.6 ± 1.7	a	1.9 ± 2.3	ab	8.3 ± 10.7	bc			
	0.0009337	**B**	**SO4**	0.0 ± 0.1	a	2.7 ± 2.4	ab	2.5 ± 2.2	ab	10.0 ± 6.6	c	1.34e-06	0.0266136	0.8698
		**A**	**R110**	0.0 ± 0.1	a	0.1 ± 0.3	a	0.2 ± 0.4	a	0.6 ± 1.1	a			
DBI (Directional Bias Index)	n.s.			**B**		**AB**		**A**		**A**		***	n.s.	n.s.
		**–**	**101-14**	−0.02 ± 0.36	–	−0.25 ± 0.20	–	−0.28 ± 0.22	–	−0.38 ± 0.33	–			
	0.1732	**–**	**SO4**	−0.01 ± 0.19	–	−0.32 ± 0.36	–	−0.51 ± 0.18	–	−0.41 ± 0.22	–	8.953e-05	0.3795	1
		**–**	**R110**	−0.10 ± 0.39	–	−0.07 ± 0.22	–	−0.22 ± 0.22	–	−0.38 ± 0.17	–			

Supplementary Table S7: Root-to-shoot relationships in grapevines grafted onto three different rootstocks (101-14, SO4, and R110) across four timepoints (T1–T4). Parameters include root-to-shoot dry weight ratio and root-to-shoot length ratio, calculated based on digitized 3D architecture and dry biomass. Values are shown as means ± standard deviation. Statistical outcomes from linear mixed-effects models reflect the effects of genotype, time, their interaction, and block. Capital letters indicate significant differences among genotypes or timepoints (*p* < 0.05) based on post hoc comparisons. Lowercase letters following numerical values denote significant genotype × time interactions where applicable.

**Parameter**	**Rootstock effect**		**Rootstock**	**T1**		**T2**		**T3**		**T4**		**Time effect**	**Interaction rootstock_time**	**Block effect**
Root to Shoot	***			**B**		**A**		**A**		**A**		***	n.s.	**
Biomass Ratio (g		**A**	**101-14**	1.6 ± 0.2	–	0.9 ± 0.1	–	0.7 ± 0.2	–	1.0 ± 0.2	–			
dryweight Root / g	2.343e-11	**A**	**SO4**	1.5 ± 0.6	–	1.0 ± 0.3	–	0.9 ± 0.3	–	1.0 ± 0.2	–	7.901e-11	0.7387	0.001687
dryweight Shoot)		**B**	**R110**	2.1 ± 0.4	–	1.4 ± 0.4	–	1.5 ± 0.5	–	1.5 ± 0.3	–			
Root to Shoot	***			**BC**		**C**		**AB**		**A**		***	n.s.	n.s.
Length Ratio (cm		**A**	**101-14**	7.1 ± 1.2	–	6.8 ± 1.5	–	4.9 ± 1.3	–	4,6 ± 0.8	–			
total root length /	6.133e-05	**B**	**SO4**	8.3 ± 2.1	–	8.3 ± 4.0	–	6.4 ± 2.1	–	5.7 ± 1.9	–	0.0009915	0.5702640	0.1082
cm total shoot		**B**	**R110**	7.8 ± 1.8	–	9.7 ± 3.3	–	8.3 ± 2.6	–	7.6 ± 1.5	–			

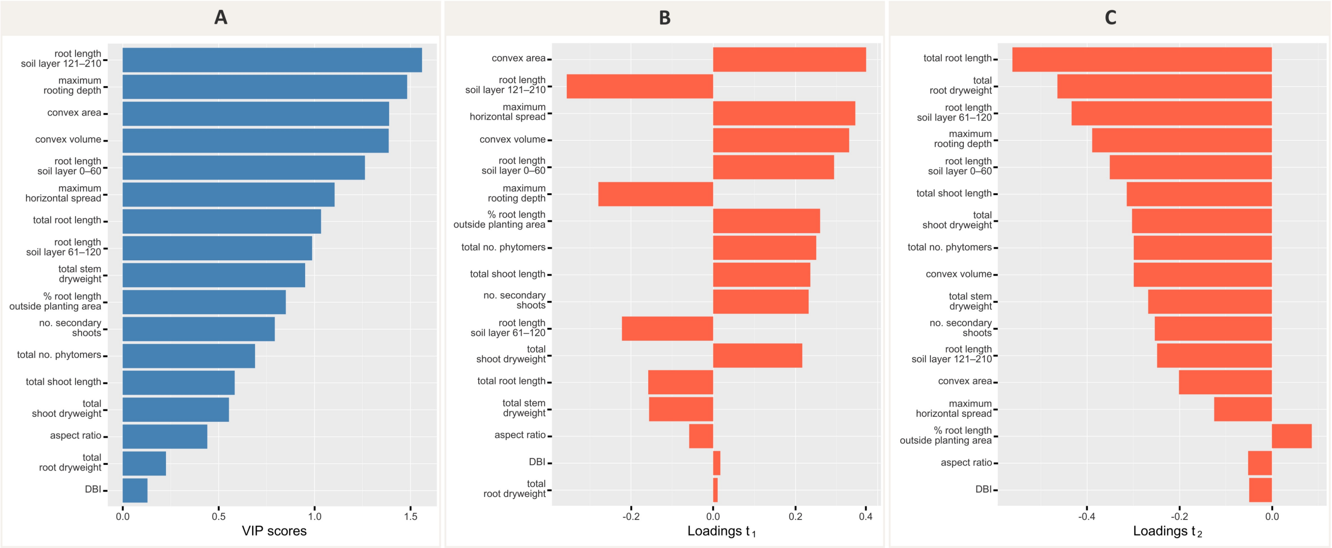

Supplementary Figure S8: Variable importance in projection (VIP) scores and loading plots from the partial least squares discriminant analysis (PLS-DA) of grapevine morphological and architectural parameters. (A) VIP scores indicating the relative contribution of each variable to genotype separation, with higher values signifying greater discriminatory power. (B) Loading plot for the first latent variable (t1), illustrating how each parameter influences separation along the t1 axis. (C) Loading plot for the second latent variable t2, showing parameter effects on the t2 axis. Negative and positive loading values reflect the direction of each variable's impact on the respective latent variable.
